# Ultraviolet-A absorbance analysis in thin porcine corneas pre-and post-crosslinking

**DOI:** 10.5935/0004-2749.2022-0273

**Published:** 2024-02-23

**Authors:** Renan Rodrigues, Paulo Schor, Bernardo Kaplan Moscovici, Priscila Guidini, Mario Antonio Stefani, Patrícia Alessandra Bersanetti

**Affiliations:** 1 Department of Ophthalmology, Universidade Federal de São Paulo, São Paulo, SP, Brazil; 2 Research & Development Department, Opto Eletronica S.A., São Paulo, SP, Brazil; 3 Department of Biochemistry, Universidade Federal de São Paulo, São Paulo, SP, Brazil

**Keywords:** Ultraviolet light, Spectrophotometry, Crosslinking, Keratoconus, Corneal thickness

## Abstract

**Purpose:**

To determine the absorbance coefficient of the thin porcine cornea to ultraviolet-A radiation (365 nm) submitted for crosslinking.

**Methods:**

This in vitro, benchtop experiment using cadaver tissue study analyzed 12 porcine corneal lamellas, which were obtained using a microkeratome after mechanical de-epithelization and separated into three thickness groups: 180, 300, and 360 µm. The corneal thickness values were measured by anterior-segment optical coherence tomography. All lamellas had ultraviolet-A (365 nm) absorbance measured with a 96-well plate spectrophotometer using an ultraviolet transparent microplate before riboflavin instillation and preand post-crosslinking according to the Dresden protocol.

**Results:**

The ultraviolet absorbance profiles of the 180, 300, and 360 µm groups were obtained as α-coefficients of 12.85, 76.55, and 120.27, respectively. A theoretical formula was calculated though a statistical analysis that demonstrated the correlation between stromal lamellar thickness and ultraviolet absorbance.

**Conclusions:**

Corneal thickness and ultraviolet-A spectral absorbance of corneal lamellas showed linear correlation. These findings can potentially contribute to the optimization of ultraviolet-A application during crosslinking, making the treatment of corneas with thickness <400 µm safe and personalized energy delivery for each corneal thickness.

## INTRODUCTION

Keratoconus is a corneal ectasia that presents with progressive corneal thinning, a typical disorder finding. To halt this behavior, crosslinking became the primary surgical option, and several studies have evaluated its safety and efficacy^(1-3)^. However, crosslinking in thin corneas (<400 µm of corneal thickness) can result in high risks of complications, mainly regarding the endothelium^(4)^. Thus, some options have become available in the last few years, including transepithelial crosslinking, contact lens-assisted crosslinking, hypo-osmolar crosslinking, custom epithelial debridement, and lenticule-assisted crosslinking^(5-8)^. Nevertheless, these are good options; however, none has similar efficacy to that of the Dresden protocol, consequently showing higher failure rates. These very thin corneas have higher risks of damaging the endothelium^(2)^. If the absorption coeffi-cient were known, we could fine-tune UV radiation delivery to crosslink the cornea and not damage the endothelium.

Hypo-osmolar crosslinking, as one of the most used options, is based on the assumption that a hydrated cornea becomes thicker, and this can prevent endothelium damage^(9,10)^. However, as previously described, hydrated corneas submitted for crosslinking showed a smaller collagen organization. This change appeared to be temporary when compared with control groups^(11)^. By contrast, even when using hypo-osmolar riboflavin, some corneas did not demonstrate a more significant increase in thickness, making it unpredictable to proceed with the procedure.

The Dresden protocol involves applying ultraviolet-A (UV-A) radiation in 365 nm, 3.0 mW/cm^2^, for 30 min, reaching 5.4 J/cm^2^, in a cornea previously saturated with riboflavin 0.1%^(2)^ for 30 min. A corneal thickness value of >400 µm at the corneal thinnest point without epithelium is the security barrier for endothelium protection, as the toxic endothelium threshold (0.35 mW/cm^2^) and the toxic threshold of total energy (0.63 J/cm^2^) were not reached^(12)^.

Thus, this study aimed to describe the UV-A absorbance pattern in thin porcine corneas before and after riboflavin instillation and post-crosslinking, creating a theoretical absorbance profile that can enable UV-A radiation delivery optimization according to corneal thickness.

## METHODS

### Corneal specimen preparation

This study examined 12 porcine thin corneal lamellas, which were obtained using a microkeratome (Moria, LSK-ONE, France) after mechanical de-epithelialization and were then separated into three thickness groups: 180, 300, and 360 µm. Immediately after the microkeratome cut, lamellas were evaluated by anterior-segment optical coherence tomography (Optovue, Fremont, EUA) for corneal thickness measurement. The diameter of all lamellas was adjusted with a trephine to fit in the 96-well plate and positioned at the bottom plate, perpendicular to the beam of light.

All porcine eyes were obtained from a slaughterhouse, examined within 6 h after enucleation, and transported in a wet chamber made with a plastic cup containing wet gauze above and below the globe.

### Crosslinking procedure

During the saturation period, an iso-osmolar riboflavin 0.1%, 400 mOsm 20% dextran (Ophthalmos, Sao Paulo, Brazil) was used, with one drop every 5 min. All lamellas had UV-A absorbance measurements at 365 nm before and after the instillation and post-crosslinking procedure. Lamella thickness was assumed constant throughout the experiment, as an isosmolar riboflavin was used.

According to the Dresden protocol, crosslinking was performed using an Opto XLink (Opto, Sao Carlos, Brazil).

### Absorbance determination

A 96-well UV transparent microplate was used to determine the UV absorbance of lamellas. Measurements were obtained using an Epoch 2 spectrophotometer (Biotech Instruments, USA). The 365-nm range used during crosslinking was selected. The analyses were performed at ambient temperature (approximately 27°C).

### Estimate of absorbance coefficient

Regarding the theoretical model used to estimate the UV absorbance coefficient of the porcine anterior corneal stroma, the Beer-Lambert law was followed. This law states that the absorbance (A) of a beam of collimated monochromatic radiation (in this case, UV-A 365 nm) in a homogenous material (anterior corneal stroma) is proportional to the absorption path length (d), in this case, corneal thickness, and to the absorbance coefficient (α), which is a characteristic of the material^(13)^. In addition, transmittance (T) is defined as the ratio of the transmitted intensity (I) over the incident intensity (I_0_) and assumes values between 0 and 1. Finally, the absorbance of the material is related to the transmittance, that is, to the incident, and transmitted intensities, through the following equations:


(equation 1) $$T=l/l_{0}=e^{-\alpha d}$$

(equation 2) $$A=-\log l/l_{0}$$

(equation 3) $$A=-\log e^{-\alpha d}=>A=\alpha d\log e$$

(equation 4) A=0.43αd

where:

A = absorbance

α = absorption coefficient (cm^-1^)

d = optical pathway (corneal thickness) (cm)

T = transmittance

I = transmitted light intensity (mW)

I_o_ = incident light intensity (mW)

e = Euler’s number

Thus, an exponential relation was found between light-transmitted intensity (I) and corneal thickness (d). In addition, a linear relation was found between absorbance (-log I/I_0_) and corneal thickness (d) since the material absorbance coefficient (α) is constant as a material characteristic.

After calculating all measures, the absorbance of each corneal lamella’s UV-A (365 nm) was obtained. The angular coefficient corresponds to the absorbance coefficient (α) through linear regression.

Finally, according to the Dresden protocol^2^, a work power value of 3 mW/cm^2^ was safe for the endothelium in corneas with stromal thickness of >400 µm. Thus,


(equation 5) $$P(e)=P(D)\times e^{-\alpha (D)}$$

(equation 6) $$P(D)\times e^{-\alpha (D)}=P(d)\times e^{-\alpha (d)}$$

(equation 7) $$P(d)=P(D)\times e^{-\alpha (D-d)}$$

where:

P (e) = maximal work power at endothelium (mW/cm^2^)

P (D) = work power according to the Dresden protocol (3 mW/cm^2^ considering D = 400 µm stromal thickness)

P (d) = work power according to corneal thickness (mW/cm^2^)

α = absorption coefficient (cm^-1^)

d = optical pathway (corneal thickness) (cm)

e = Euler’s number

After the α constant was determined, the optimized work power was calculated according to each corneal thickness, using the following equation:


(equation 8) $$P(d)=3\times e^{-\alpha (0.04-d)}$$

Therefore, this final equation can make it possible to correlate corneal thickness and work power on the crosslinking device .

## RESULTS

This study revealed a linear correlation between corneal thickness and corneal UV-A (365 nm) absorbance (A) in corneal lamellas previously soaked with riboflavin.

All lamellas had UV-A absorbance measured by a spectrophotometer preand post-riboflavin soaking and post-crosslinking, and the results are presented in table 1. The mean corneal thickness in the 180, 300, and 360 µm groups were 219.50, 322, and 377 µm, respectively. As intended, each thickness value was analyzed individually and not as a group to obtain results for every sample with different thickness values, with a linear and progressive character, from the thinnest to the thickest lamella, which, ultimately, would optimize results. The biomechanical effects of crosslinking are already widely known, so they were not the focus of this study, which aimed to identify changes in the absorption of UV energy by the tissue.

**Table 1:** Total UV-A absorbance (365 nm) of lamellas in all groups, without microplate absorbance. Pachymetry values are presented in cm

Without riboflavin		With riboflavin		Post CXL	
**Pachymetry**	**Absorbance (A)**	**Pachymetry**	**Absorbance (A)**	**Pachymetry**	**Absorbance (A)**
0.0196	0.1647	0.0196	0.8267	0.0196	1.1197
0.0219	0.2117	0.0219	0.6837	0.0219	1.3217
0.0223	0.1297	0.0223	0.6777	0.0223	1.0827
0.0240	0.1557	0.0240	0.8847	0.0240	1.2787
0.0308	0.1157	0.0308	1.0857	0.0308	1.3747
0.0316	0.1567	0.0316	1.0647	0.0316	1.6807
0.0328	0.1177	0.0328	1.2447	0.0328	1.8867
0.0336	0.1727	0.0336	1.0637	0.0336	1.5677
0.0365	0.2007	0.0365	1.1817	0.0365	1.8337
0.0376	0.1817	0.0376	1.1787	0.0376	1.8507
0.0379	0.2607	0.0379	1.1367	0.0379	2.0027
0.0388	0.2157	0.0388	1.1847	0.0388	2.0857

**Figure 1:**
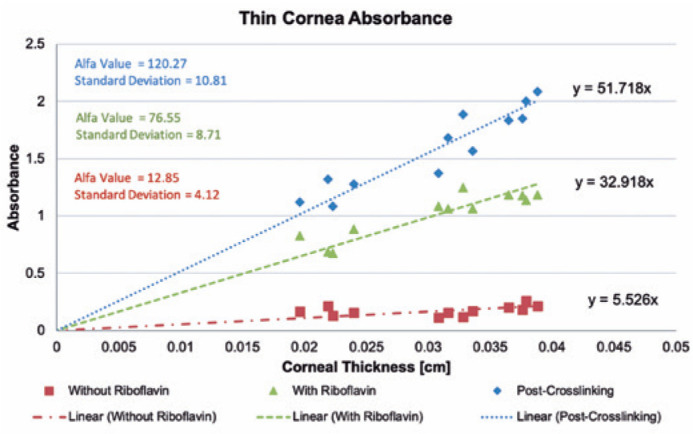
Absorbance of thin porcine cornea and alpha values.

Pre-soaking lamellas had a lower absorbance variability, with some thinner lamellas absorbing even more energy than thicker lamellas in a similar range. This group showed a weak linear correlation between corneal thickness and UV absorbance (Figure 1).

A linear correlation was found in the post-soaking lamellas because thicker lamellas showed a higher UV absorbance profile than thinner lamellas. Moreover, the post-crosslinking samples had an even higher correlation (Figure 1). This linear correlation was achieved because of the exponential behavior reduced by the log. That is, although the absorbance ratio was linear, the power ratio that can be safely applied will be logarithmic. The microplate was also tested for UV absorbance, showing a low value of 0.043, considered in all calculations.

After this, the α-coefficient was determined for each group according to the previously mentioned equations, and the results are presented in figure 1. The pre-soaking, post-soaking, and post-crosslinking groups showed 12.85, 76.55, and 120.27, respectively. At this point, linear regression calculated how all groups absorbed UV-A, according to each corneal thickness (Figure 1).

Finally, the post-riboflavin coefficient was used to calculate the optimized work power because at this moment UV irradiation starts during the crosslinking procedure (Table 2). With this optimization, for each corneal thickness, suggesting a working power that theo-retically will not reach the toxic endothelial threshold was already possible.

**Table 2:** Work power according to pachymetry using post-riboflavin alpha value

Pachymetry (cm)	Estimated endothelium Power (mW)	Work power (mW)
0.0196	0.073423349	0.629342992
0.0219	0.087559189	0.750507338
0.0223	0.090281847	0.773844403
0.0240	0.102830021	0.881400177
0.0308	0.173060468	1.483375443
0.0316	0.183990450	1.577060997
0.0328	0.201693262	1.728799388
0.0336	0.214431605	1.837985184
0.0365	0.267734534	2.294867435
0.0376	0.291256634	2.496485435
0.0379	0.298023049	2.554483277
0.0388	0.319280162	2.736687104

## DISCUSSION

Thinner lamellas had a lower absorbance profile than thicker lamellas, and the proposed theoretical model was made ex vivo using porcine corneas. Therefore, through energy optimization, the barrier of 400 µm of corneal stromal thickness for crosslinking can be overcome^(2)^.

The pre-soaking group demonstrated a lower varia-bility, with thinner corneas absorbing sometimes more UV-A energy than thicker corneas, even in low order. This was possibly explained by the lower absorbance coefficient of the corneal stroma to the UV-A radiation^(13,14)^, mainly in thin lamellas, which makes less difference when analyzing all group thicknesses. Post--soaking and post-crosslinking groups showed a linear correlation between these two variables.

This study used iso-osmolar riboflavin 0.1% (400 mOsm), and crosslinking is possible with hypo-osmolar riboflavin (310 mOsm), which can induce corneal edema, increase thickness, and protect the endothelium^(9,10)^. However, this strategy may not be adequate to prevent progression^(15)^. Transepithelial crosslinking^(5)^, custom epithelial debridement at the cone apex^(6)^, and contact lens-assisted crosslinking^(7)^ may also halt progression on thin corneas^(16)^. Therefore, none of them emphasize the main problem, which is the higher energy delivery to the cornea, which exceeds the toxic endothelium threshold. Moreover, these alternatives have higher failure rates than the Dresden protocol, which is still the gold standard. The use of human corneal lamellas from smile procedures is another option^(8)^; however, a corneal transplant technique involves tissue rejection risks and depends on the availability of technology and donors.

In another study, porcine corneas submitted with smaller intensities of UV-A (1.5 mW/cm^2^) exerted a biomechanical stiffening effect similar to that of the Dresden protocol. This finding also supports the idea that UV-A energy delivery can be reduced, possibly maintaining biochemical, and clinical crosslinking effects, even in corneas thinner than 400 µm^(17)^. Therefore, for these extreme cases reducing fluence and irradiance during crosslinking can be an option.

Hafezi et al. reported another alternative for the treat-ment of thin corneas^(18)^. They proposed the “sub-400 protocol”, in which, using a theoretical formula, the fluency of the UV energy needed for each patient was estimated according to corneal thickness. However, in their formula, they kept the work power constant at 3 mW/cm^2^, varying the time of exposure to UV energy; however, this study proposes to keep the time constant, varying the work power applied to the cornea. Since studies have already prove the production of crosslinking in scenarios with less work power, this other approach is reasonable for future comparative works^(17,18)^.

This study has the following limitations: relatively small number of samples and mechanical creation of lamellas (instead of femtosecond-created ones), which increased thickness variability. The use of four lamellas in each group, instead of the intended three, was an attempt to improve accuracy regarding this possible variability when performing mechanical LASIK. Conversely, this study evaluated the main barrier to crosslinking thinner corneas and the higher amount of energy delivered and proposed a viable alternative that can be further explored.

Further complementary studies are needed to evaluate this energy optimization in vivo on thin corneas and analyze histopathological, biochemical, and biomechanical behaviors. Owing to the reduction of energy delivery according to corneal thickness, the potential harms of this strategy are minimized because the toxic endothelial threshold was not reached. Although the maximum energy level to perform crosslinking is already known, the minimum energy required for each patient is still being investigated, and reducing this energy at lower levels can be a solution to reduce the incidence of some crosslinking complications, such as corneal haze, progressive flattening, persistent epithelial defects, and endotheliitis, which can be related to the amount of energy delivered^(19)^.

Several crosslinking instruments are currently available, and many already have control over energy optimization, which could facilitate the adoption of this irradiance modification, as they would be ready for use^(20,21)^.

Finally, the results of this study revealed a linear correlation between porcine corneal thickness and UV-A absorbance. Through this theoretical model, the amount of energy each corneal thickness can support during crosslinking can be calculated, making personalized treatment for each thickness possible.
